# Failure Analysis of Girth Weld Cracking in Gas Transmission Pipelines Subjected to Ground Subsidence and Traffic Loads

**DOI:** 10.3390/ma17225495

**Published:** 2024-11-11

**Authors:** Lifeng Li, Xiangzhen Yan, Lixia Zhu, Gang Wu, Shuxin Zhang

**Affiliations:** 1 College of Pipeline and Civil Engineering, China University of Petroleum, Qingdao 266580, China; 2 State Key Laboratory of Performance and Structural Safety for Petroleum Tubular Goods and Equipment Material, CNPC Tubular Goods Research Institute, Xi’an 710077, Chinazhangshuxin003@cnpc.com.cn (S.Z.)

**Keywords:** girth weld crack, failure analysis, subsidence, weld quality

## Abstract

Girth welds are weak points in pipelines, and failures occur frequently. In a gas transmission pipeline, a girth weld experienced cracking, prompting a failure analysis using experimental methods and finite element analysis (FEA). Experimental results showed that X-ray non-destructive testing (NDT) revealed cracks, porosity, and lack of fusion in the girth weld. However, the hardness and microstructure of the material showed no abnormalities. During operation, the pipeline experienced an increase in soil cover and was subjected to ground subsidence and vehicle loads. Finite element analysis was conducted on the defective girth weld under different conditions, including varying soil cover depths, different levels of subsidence, and varying vehicle loads, to examine the pipeline’s stress response. The results indicated that the combination of soil cover, subsidence, and vehicle loads led to pipeline failure, whereas none of these factors alone was sufficient to cause girth weld failure. To prevent such failures from occurring again, the following measures are recommended: strengthen on-site welding quality control of girth welds, conduct inspections for defects in girth welds of in-service pipelines, and promptly address any defects that exceed acceptable limits.

## 1. Introduction

Long-distance oil and gas pipelines offer several advantages, including low transportation costs, minimal losses, reduced permanent land use, fast construction speed, large transport capacity, and high safety [1]. These benefits have made them a widely adopted transportation method for oil and gas resources by energy companies worldwide, especially in China, where pipeline construction is rapidly developing [2]. However, in the event of failure in high-grade steel, high-pressure, and large-diameter pipelines, the impact radius [3] can extend several hundred meters, potentially causing casualties and property damage. In recent years, there have been several incidents of pipeline circumferential weld seam failures in the world, resulting in significant losses.

Both domestic and international pipelines face the issue of girth weld failure. According to PHMSA reports [4], multiple large-diameter (≥508 mm), high-grade (×70 or higher) natural gas and hazardous liquid pipelines constructed between 2008 and 2009 experienced leaks and fractures due to girth weld failures during pressure testing or operation [5]. Post-incident failure analysis revealed that most of these girth weld failures were related to factors such as misalignment [6], substandard machining of bevels for varying wall thicknesses, poor root welding, and inadequate pipeline support. PHMSA recommended that pipeline operators comprehensively assess the risk of girth weld failure in newly constructed large-diameter pipelines, conduct necessary engineering fitness-for-service evaluations, and control operating pressures on pipelines with identified issues. Wang et al. [7,8,9] conducted extensive research on the issue of weld misalignment. Their studies indicate that misalignment magnitude, weld strength mismatch, and weld profile significantly impact weld performance. If the weld profile is appropriate, even in cases of substantial misalignment, the load-bearing capacity is minimized to the lowest possible reduction. Welding causes the metal to melt, with the cooling rate influencing the microstructure of the weld and the heat-affected zone. The presence of hard and brittle phases, such as martensite, increases the likelihood of cracking and failure under stress concentration [10,11,12,13]. Additionally, defects like porosity and slag inclusions can compromise the mechanical properties of the weld, potentially leading to failure under excessive load. The Canadian Energy Regulator (CER) [14] summarized common problems with girth welds as follows: (1) the actual strength of the girth welds is lower than that of the base material [15]; (2) softening of the heat-affected zone and root softening [16]; and (3) external bending loads.

In addition to defects and external loads, the impact of materials on the failure of girth welds in high-grade pipelines has become more prominent [17]. The traditional view holds that defects, such as lack of fusion, incomplete penetration, and undercutting, are the main causes of failure. During pressure testing, several incidents of weld cracking were caused by hazardous defects in girth welds. With the use of large-diameter, high-grade pipelines, stress concentration caused by defects leads to girth weld cracking under relatively low external loads. In June 2014 and January 2018, two girth weld cracking incidents occurred in an ×70 steel gas pipeline operated by Malaysia’s Petronas in an area with frequent geological activity [18]. The investigation found that the girth welds exhibited low matching strength. To assess the load-bearing capacity of the pipeline during displacement, Petronas commissioned DNV to conduct two full-scale bending tests. The results indicate that under weak matching conditions, the maximum strain was only 0.48%, while strong matching girth welds achieved strains exceeding 3%, fully verifying the negative impact of weak matching on load-bearing capacity [19].

At present, the failure of girth welds is primarily focused on weld defects or external loads, while failure cases involving the coupling of weld defects with subsidence [20,21,22] and vehicle loads [23,24,25] are relatively rare.

**Figure 1:**
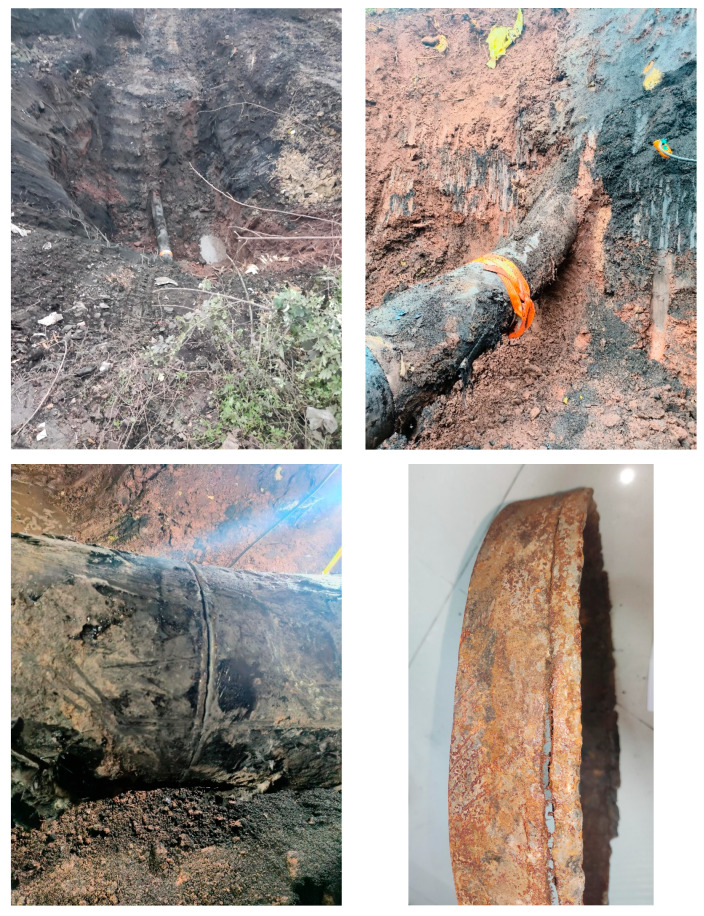
Field photos of girth weld failure.

During the operation of a certain pipeline, abnormal drops in operating pressure and production were detected. Further investigation revealed a natural gas leak. The leaked pipe was constructed in July 1986, and the pipeline utilized Φ426 mm × 9(10) mm seamless 20# steel pipes. Prior to 2000, it transported sulfur-containing wet gas, and since then, it has been transporting sulfur-containing dry gas. The operating pressure is 4.75 MPa, with a gas transmission volume of approximately 1.08 million cubic meters per day and a hydrogen sulfide content of around 7749 mg/m^3^. The leak was located between the 10 o’clock and 2 o’clock positions on the girth weld. The failure site and fracture morphology are shown in Figure 1. After excavating the pipeline, it was found that the leak was caused by a crack in the girth weld. This weld is located at the junction between an elbow (4.0D, 29 degrees) and a straight pipe, approximately 10 m from a nearby road. The depth of the pipeline at the failure location was approximately 6 m (the design burial depth during pipeline construction was 1 m). The failure morphology of the girth weld indicates that the crack propagated along the weld appeared relatively flat. Due to the size limitations of the girth weld sample provided, tensile performance tests, impact toughness tests, and others could not be conducted. Only hardness testing and metallographic analysis were performed. Additionally, based on pipeline construction documentation and on-site investigation, considering the external loads applied to the girth weld before and after the fracture, a nonlinear contact pipe–soil interaction model was used to analyze the mechanical response of the pipeline girth weld before and after the fracture using Abaqus finite element software (version 6.14). Based on the experimental and simulation results, the weld material integrity and the pipeline’s stress response under combined loading were analyzed. The failure causes were identified, and preventive measures were proposed.

## 2. Experiment

### 2.1. Non-Destructive Test

**Figure 2:**
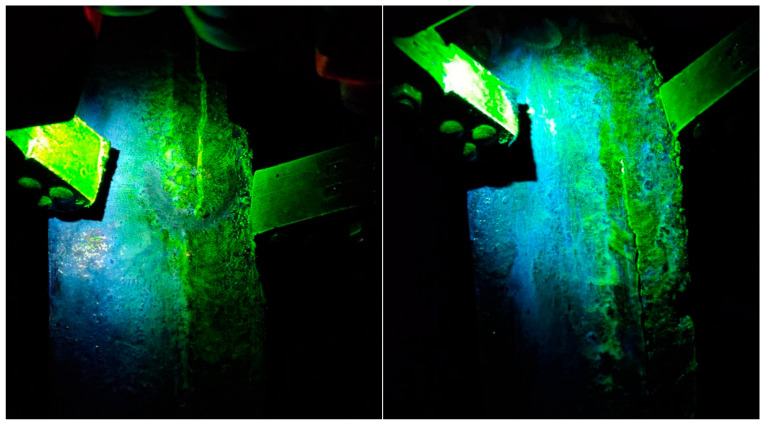
Magnetic particle inspection of circumferential welds.

A magnetic particle inspection device was used to perform magnetic particle testing on the failed weld sample in accordance with ASTM E709:2021 [26]. The results were shown in Figure 2, which showed that there are multiple surface cracks in the weld under ultraviolet fluorescent lighting.

**Figure 3:**
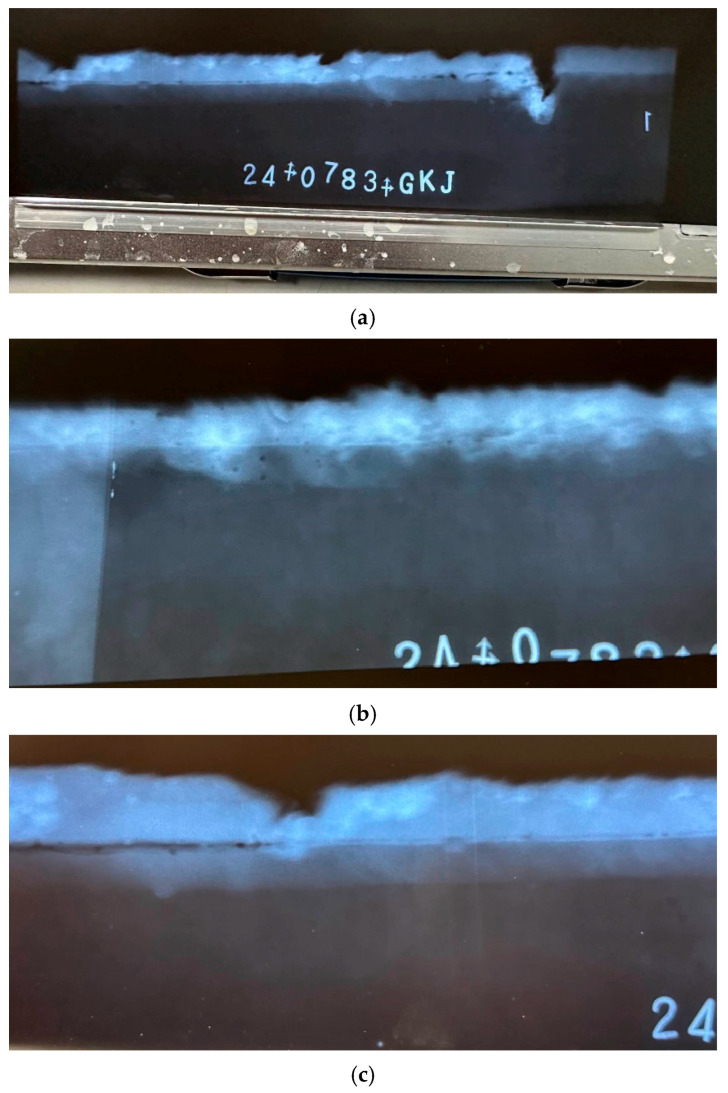
Radiographic inspection results of girth welds. (**a**) Crack and lack of fusion; (**b**) Porosity; (**c**) Lack of fusion.

A radiographic testing machine was used to perform radiographic inspection on the unbroken sections of the submitted girth weld to inspect whether there is the presence of porosity and internal cracks, in accordance with ASTM E94/E94M-22 [27]. The results indicated the presence of cracks, porosity, and lack of fusion defects in the unbroken sections of the weld. Among these, the maximum crack length was 75 mm, and the maximum lack of fusion defect length was 70 mm, as shown in Figure 3. Although the pipeline was constructed in 1986, there was no requirement at that time for 100% radiographic and ultrasonic inspection of welds. In this evaluation, the original welds were assessed using the latest standards. The inspection results indicate that the girth weld does not meet the requirements of GB/T 31032-2023 [28].

### 2.2. Hardness Test

**Figure 4:**
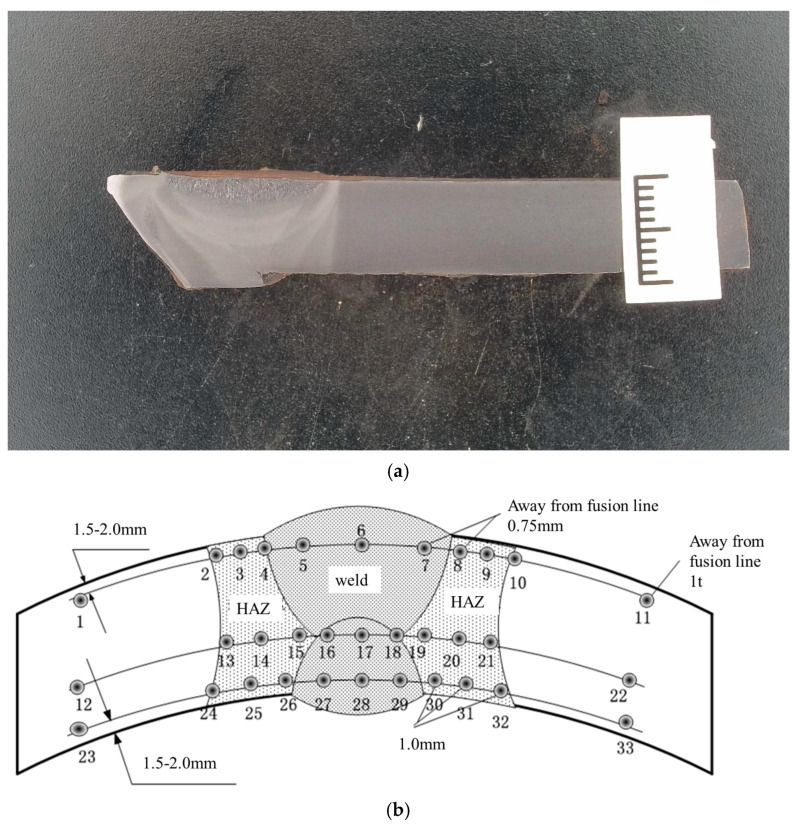
Hardness test sample and diagram of hardness test. (**a**) Hardness test sample; (**b**) Diagram of hardness test.

Samples were taken from the uncracked section of the girth weld, and Vickers hardness testing was performed using a KB30BVZ-FA hardness tester (KB Prüftechnik GmbH, Im Weichlingsgarten, Germany) according to ASTM E92-17 [29], with a test load of 10 kg. The test locations are shown in Figure 4, and the results are presented in Table 1. The average hardness in the weld area was 148 HV10, the average hardness in the heat-affected zone was 163 HV10, and the average hardness in the base material was 146 HV10. According to the hardness requirements specified in GB/T 31032-2023 [28], the hardness results of the failed weld meet the required standards.

### 2.3. Microstructure

**Figure 5:**
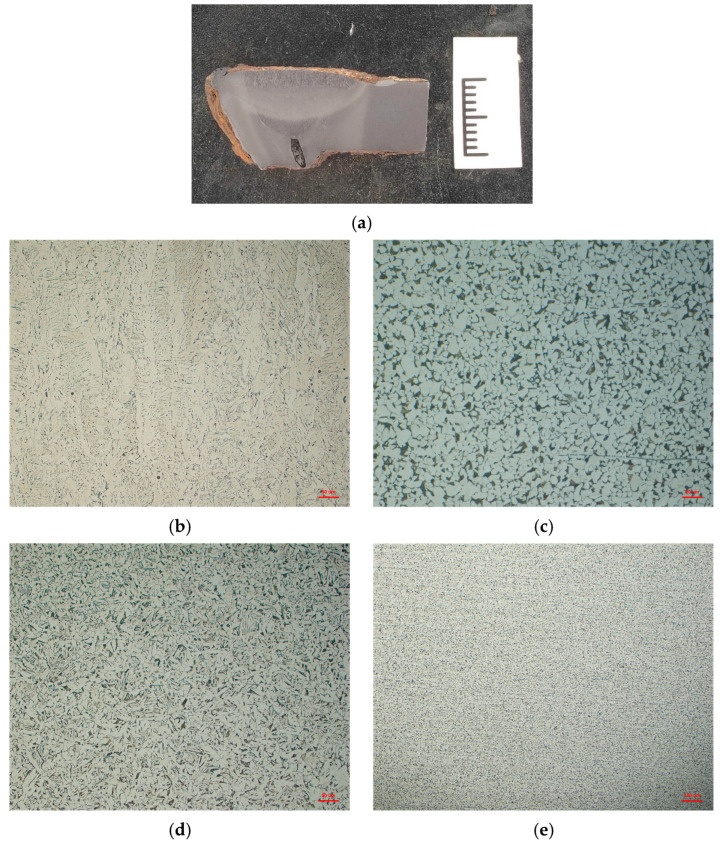
Microstructure of the girth weld. (**a**) macroscopic picture of weld; (**b**) Cap weld microstructure; (**c**) Root weld microstructure; (**d**) Heat affected zone coarse grain microstructure; (**e**) Fine grain microstructure.

The microstructure and cracks at the failed girth weld were observed using an OLS 4100 laser confocal microscope (Olympus, Tokyo, Japan). The microstructure analysis results are shown in Figure 5. No obvious microstructural abnormalities or abnormal grain growth were observed in the weld and heat-affected zone.

**Figure 6:**
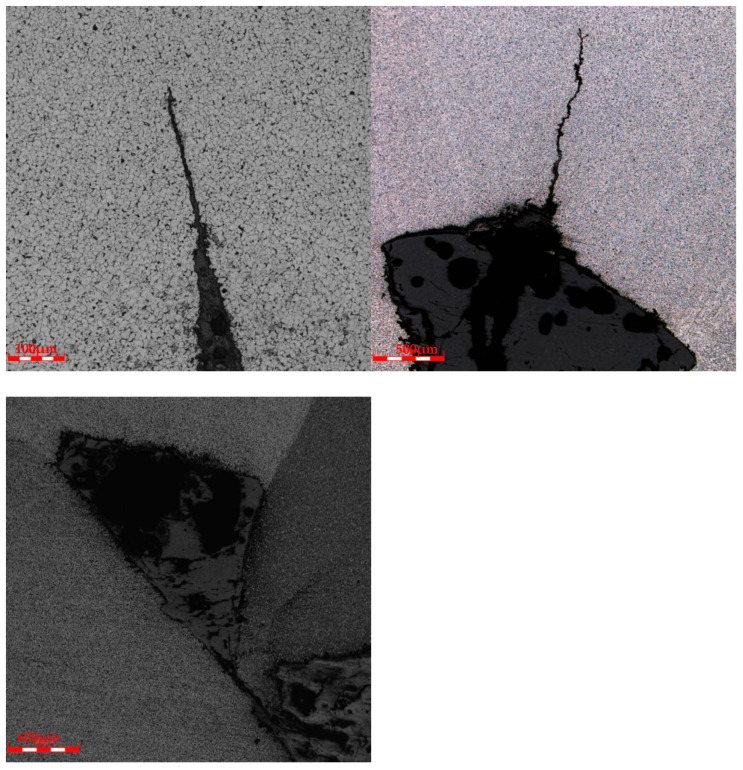
Cracks at the weld toe of the sample and grayish material.

**Figure 7:**
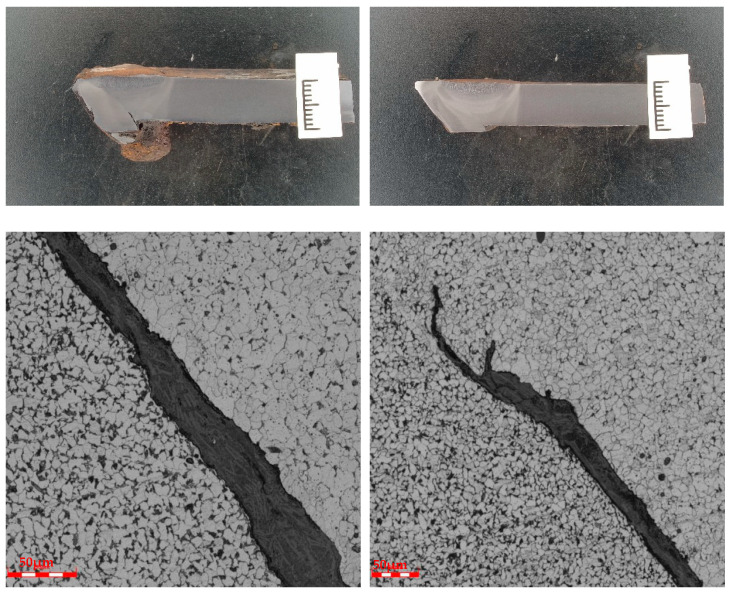
Morphology of crack propagation along the grayish material in the sample.

Cracks were observed in the failed girth weld, as shown in Figure 6 and Figure 7. The cracks originated at the weld toe on the inner surface of the pipe and extended toward the weld fusion zone. A grayish material was observed inside the cracks.

### 2.4. Scanning Electron Microscope Analysis of Girth Welds

**Figure 8:**
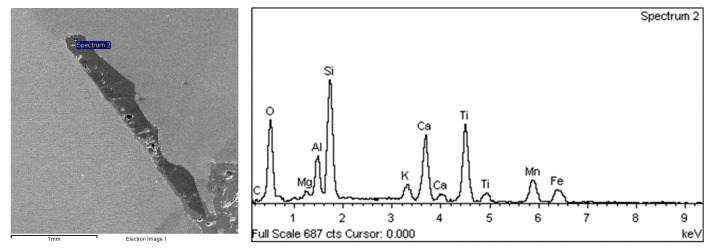
Position and spectrum of the energy dispersive spectroscopy (EDS) analysis of the grayish material.

Observations of grayish material in the crack area were carried out using scanning electron microscopy and its chemical composition was carried out with the use of EDS. The results are shown in Figure 8. The primary chemical elements in the grayish material include O, Si, Ti, Ca, and Al. The elements O and Si may originate from the sandpaper used during the preparation of the metallographic sample, while the presence of Ti, Ca, and Al indicates that the material mainly consists of welding slag.

## 3. Finite Element Analysis

**Figure 9:**
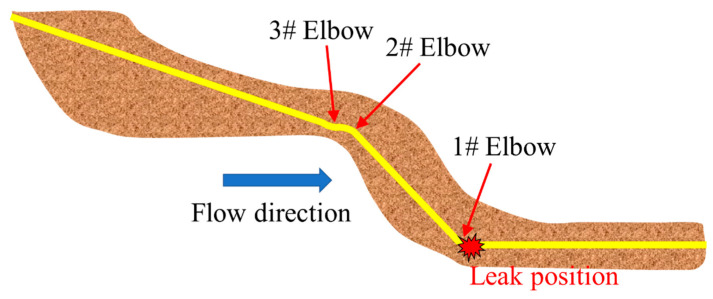
Schematic diagram of the girth weld failure location on the pipeline.

**Figure 10:**
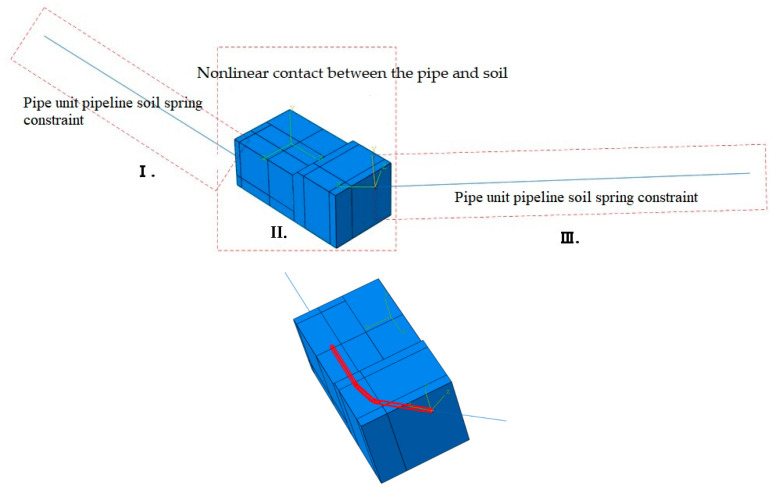
Overview diagram of the model.

**Figure 11:**
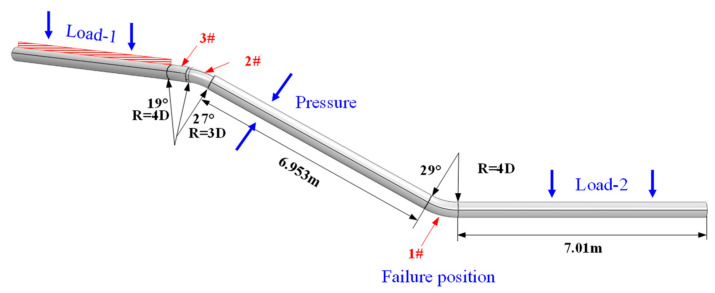
Schematic diagram of the pipeline model, 1#, 2#, 3# indicate 3 bend in the failed pipe.

**Figure 12:**
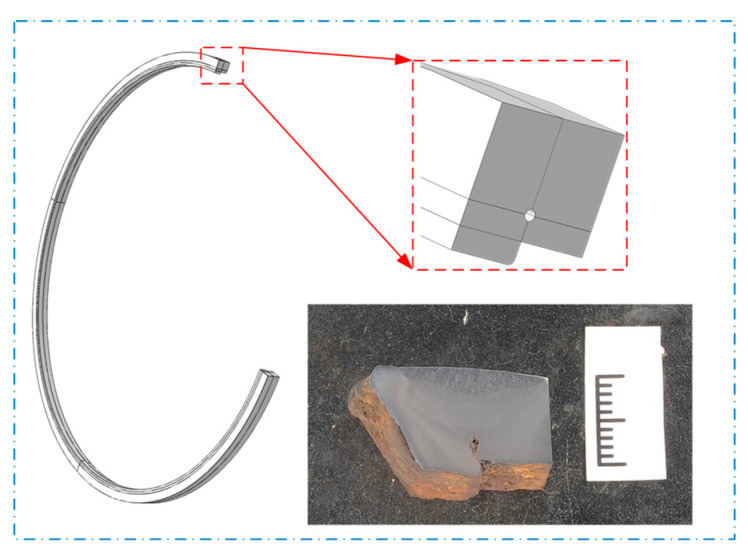
Schematic diagram of the girth weld with varying wall thickness.

Based on finite element analysis, a pipe–soil geometric model was constructed to simulate the field conditions (Figure 9). An overview of the model is shown in Figure 10 and Figure 11. In this model, Sections I and III represent the pipe segments outside the region of girth weld fracture analysis. The pipe–soil interaction is modeled using soil springs, aimed at reducing boundary effects in the solid model and preventing inaccurate calculations of mechanical parameters of the pipeline. Section II represents the nonlinear contact geometric model between the pipe and soil. The pipeline has a diameter of 426 mm, with the elbow section having a wall thickness of 10 mm and the straight pipe section having a wall thickness of 9 mm. Figure 12 illustrates the girth weld at the fracture location, where the wall thickness varies. The welding defect is represented by a circular pore with a diameter of 0.1 mm, distributed circumferentially along the pipeline.

### 3.1. Mesh

Sections I and III of the pipelines each have a length of 50 m. The mesh is applied at 1 m intervals, resulting in a total of 50 elements. These sections are constructed using three-dimensional Pipe31 elements, with the cross-sectional dimensions matching those of Section II. Surrounding the pipeline, soil springs are added at 1 m intervals, providing resistance in the lateral, axial, vertical upward, and vertical downward directions. The stiffness of these springs is determined based on the calculation methods provided in the “Guidelines for the Design of Buried Steel Pipe”.

**Figure 13:**
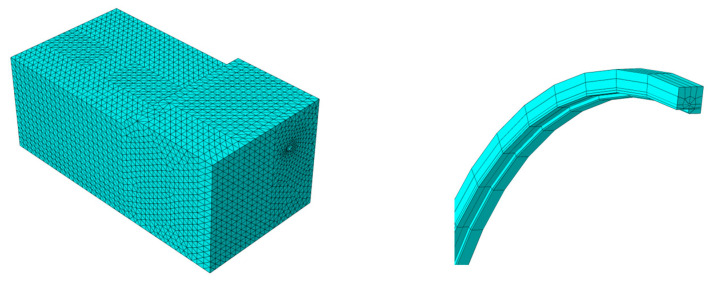
Mesh division of Section II.

Three-dimensional solid models of the soil and pipeline were constructed using C3D8R elements, as shown in Figure 13. The soil portion was meshed using a free tetrahedral division method, with mesh nodes spaced at 0.6 m intervals, resulting in a total of 103,821 elements. The pipeline was meshed using a hexahedral structure, with mesh nodes spaced at 0.35 m intervals. Mesh refinement was applied at the girth weld and elbow sections, resulting in a total of 33,888 elements.

### 3.2. Materials Parameter

**Figure 14:**
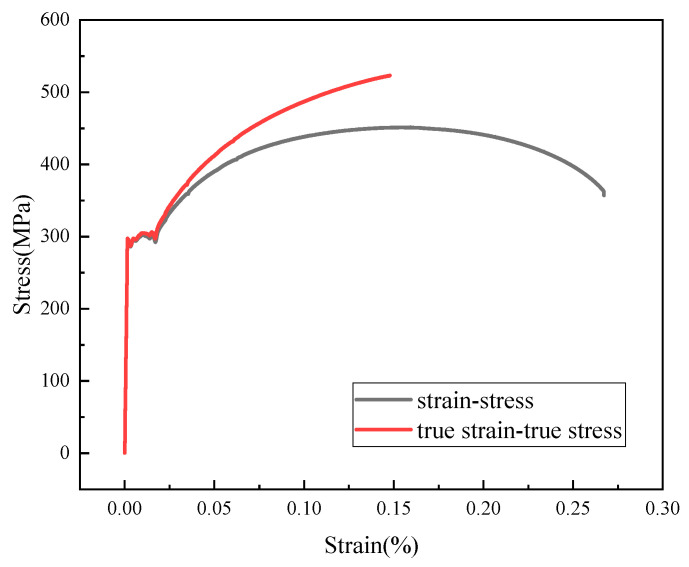
Tensile stress–strain curve of 20# steel.

The pipeline material is 20# steel, with a density of 7850 kg/m^3^, an elastic modulus of 210 GPa, and a Poisson’s ratio of 0.3. The elastic–plastic constitutive model uses the true stress–strain curve, which was derived from the uniaxial tensile test data for 20# steel, as shown in Figure 14.

Due to the lack of mechanical property testing of the site soil, the mechanical properties of conventional clay were selected. The clay has a density of 1600 kg/m^3^, an elastic modulus of 10 MPa, a Poisson’s ratio of 0.27, an internal friction angle of 30°, a dilation angle of 2°, and a cohesion of 3 kPa. The elastic deformation of the soil is described using a linear elastic model, while the ideal elastic-plastic deformation of the soil is modeled using the Mohr–Coulomb constitutive model.

### 3.3. Model Constraint and Load

Load: Based on field surveys and monitoring data, the external load environment of the gas transmission pipeline during its fracture includes the internal pressure of the operating pipeline, the gravity load of the overburden soil, the load from vehicles on the bridge, and ground subsidence loads monitored by InSAR. The specific settings are as follows:

(1) Internal Pressure: A pressure of 4.8 MPa is applied based on operational records.
(2) Overburden Soil Load: The gravity load of the soil is applied, with the thickness of the overburden soil set to 1 m (the thickness at the time of construction and commissioning) and 6 m (the thickness at the time of failure due to accumulation).
(3) Vehicle Load: The vehicle load is considered based on the methods provided in the references “Load Calculation for Buried Pipelines Crossing Highways” and “Buried Rigid Pipes: Structural Design of Pipelines.” A heavy vehicle load is represented by a six-axle trailer, with a total bridge vehicle load of 49 tons. Using the Spangler method, the load is set to P = 218,584 Pa. For a regular vehicle, with a total bridge vehicle load of 10.4 tons, the load is set to P = 46,545 Pa.
(4) Ground Subsidence Load: A ground surface subsidence displacement of 20 mm is applied, based on InSAR monitoring data.

**Figure 15:**
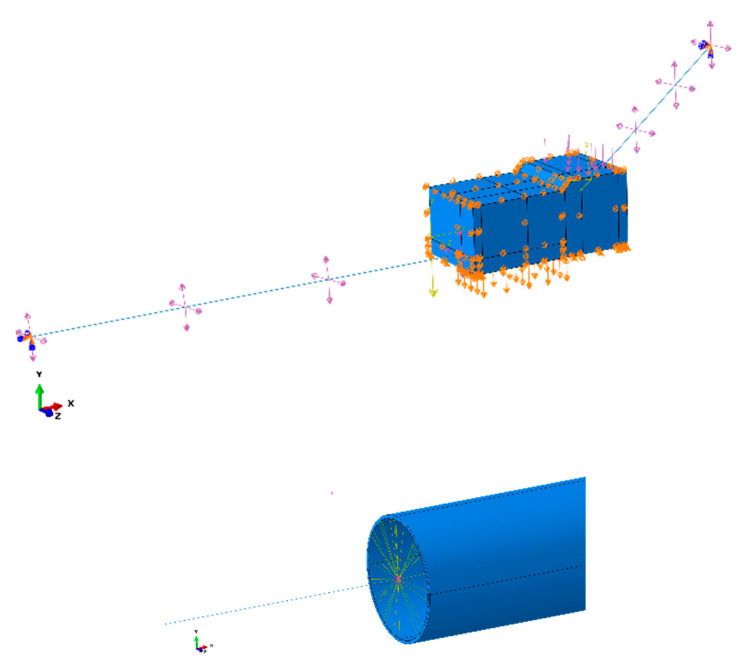
Schematic diagram of model constraint settings.

Model Constraints: As shown in Figure 15, the pipeline at both ends of the model is constrained by soil springs, so no additional constraints are necessary. Full constraints are applied to the nodes at both ends of the pipeline. For the central soil and pipeline, which are of particular interest, the four sides of the soil are constrained in the normal direction, and the bottom of the soil is fully constrained. The central pipeline is not subjected to any additional constraints. The connection between the C3D8R pipeline and the PIPE31 pipeline is fully coupled in all degrees of freedom to accurately replicate the real pipeline constraint environment.

#### Calculation Scenarios

Due to the numerous external loads present during the failure of the pipeline girth weld, a controlled variable method was used for the study. A calculation table for different working conditions was designed, as shown in Table 2. The specific working conditions are as follows:

Scenario 1: This scenario aims to elucidate the mechanical state of the pipeline at a 6 m burial depth without considering vehicle loads or subsidence loads.

Scenario 2: This scenario seeks to explain the mechanical state of the pipeline at the moment of girth weld failure, excluding subsidence loads, with a focus on the effect of a heavy vehicle load (total bridge vehicle load of 49 tons).

Scenario 3: This scenario attempts to clarify the mechanical state of the pipeline at the moment of girth weld failure, excluding subsidence loads, with a focus on the effect of a standard vehicle load (total bridge vehicle load of 10.4 tons).

Scenario 4: This scenario aims to illustrate the mechanical state of the pipeline at the moment of girth weld failure under all loads, including a standard vehicle load (total bridge vehicle load of 10.4 tons).

Scenario 5: This scenario seeks to elucidate the mechanical state of the pipeline before the overburden was accumulated, with a 1 m burial depth, excluding vehicle and subsidence loads.

Scenario 6: This scenario attempts to clarify the mechanical state of the pipeline before the overburden was accumulated, considering vehicle loads but excluding subsidence loads, with a 1 m burial depth and a standard vehicle load (total of 10.4 tons).

Scenario 7: This scenario aims to elucidate the mechanical state of the pipeline before the overburden was accumulated, excluding vehicle loads but considering a subsidence load of 0.02 m, with a 6 m burial depth.

### 3.4. Failure Criterion

Since the failed pipeline is made of 20# steel, which is classified as a low-strength steel, stress-based design criteria are typically used in the early design stages. Therefore, for evaluating the girth weld fracture failure in this case, the Mises stress state failure criterion and the Tresca stress state failure criterion, both of which are stress-based, were selected. The pipeline is considered to have failed if the stress state exceeds 0.9 times the yield strength, which corresponds to 265 MPa.

### 3.5. Calculation Results

**Figure 16:**
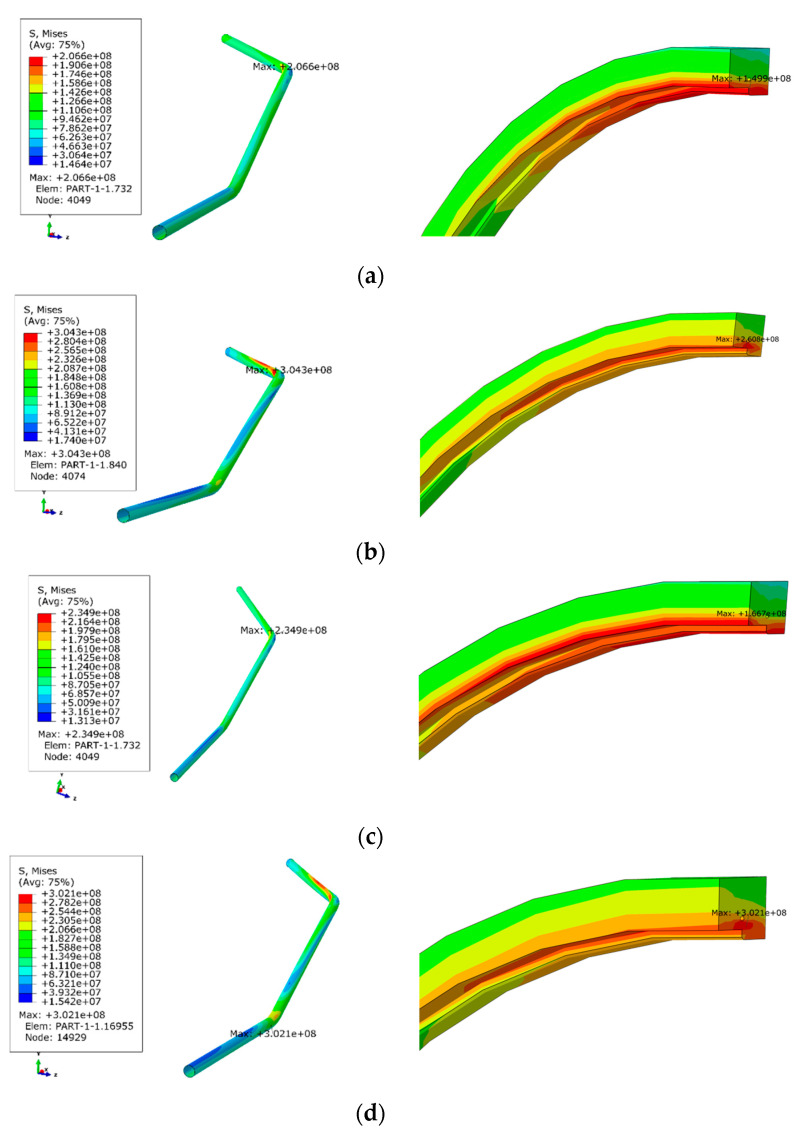

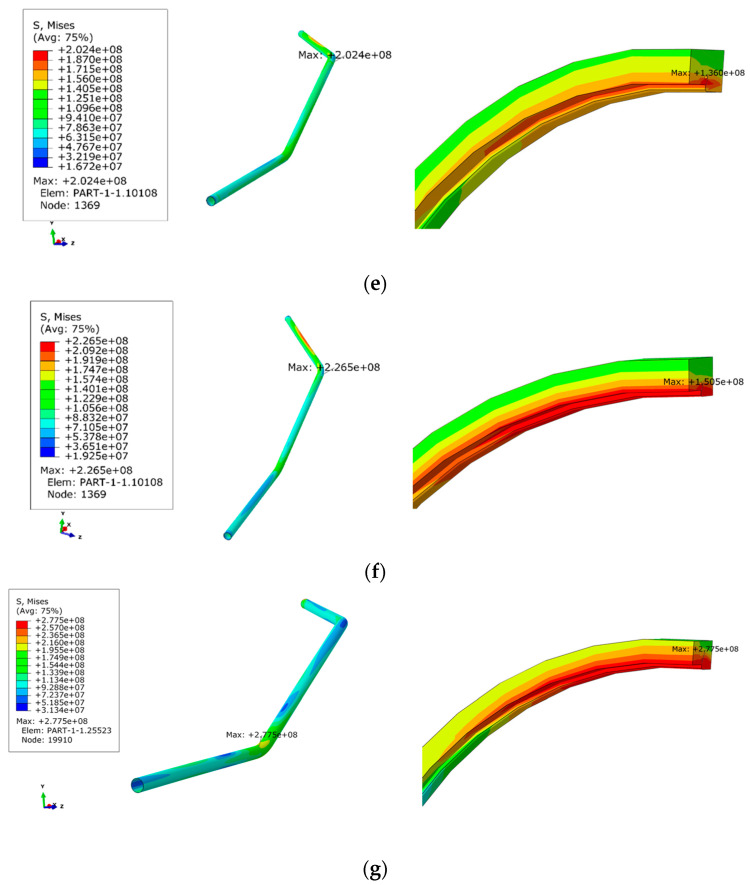
Stress distribution diagrams of the pipeline and girth weld under different calculation scenarios. (**a**) Scenario 1; (**b**) Scenario 2; (**c**) Scenario 3; (**d**) Scenario 4; (**e**) Scenario 5; (**f**) Scenario 6; (**g**) Scenario 7.

Figure 16 and Table 3 shows the stress response results of the pipeline under different installation environments, including an analysis of the maximum pipeline stress and the maximum stress at the circumferential welds.

### 3.6. Result Discussion

The stress distribution contour maps for the girth weld indicate that stress concentration consistently occurs near the 12 o’clock position of the girth weld.

Without considering vehicle or settlement loads (comparison between scenario 1 and scenario 5), when the soil thickness increases from 1 m to 6 m due to long-term accumulation on the left side of the bridge, the maximum stress in the pipeline system increases from 202.4 MPa to 206.6 MPa and the maximum stress at the girth weld increases from 136 MPa to 149.9 MPa. The pipeline’s maximum stress increases by 2.3%, while the maximum stress at the girth weld increases by 10.1%.

Considering a 10.4-ton vehicle load on the bridge without subsidence loads (comparison between scenario 3 and scenario 6), the maximum stress in the pipeline system increases from 226.5 MPa to 234.9 MPa, and the maximum stress at the girth weld increases from 149.9 MPa to 166.7 MPa as the overburden thickness increases from 1 m to 6 m. The pipeline’s maximum stress increases by 3.7%, while the maximum stress at the girth weld increases by 10.7%.

Therefore, regardless of whether vehicle loads are considered, as long as subsidence loads are not considered, increasing the overburden thickness from 1 m to 6 m results in only minor changes in the maximum stress at both the pipeline and the girth weld, and the pipeline remains in a safe state.

With a 6 m overburden and no subsidence load, the addition of a 10.4-ton standard vehicle load increases the pipeline’s maximum stress from 206.6 MPa to 234.9 MPa, and the maximum stress at the girth weld increases from 150.5 MPa to 166.7 MPa. The pipeline’s maximum stress increases by 13.7%, while the maximum stress at the girth weld increases by 11.2%, remaining within a safe range.

When the standard vehicle is replaced with a heavy vehicle load of 49 tons, the pipeline’s maximum stress increases from 234.9 MPa to 304.3 MPa and the maximum stress at the girth weld increases from 166.7 MPa to 302.1 MPa. The pipeline’s maximum stress increases by 30%, while the maximum stress at the girth weld increases by 24%, indicating that the stress levels exceed the safe threshold.

Therefore, with a 6 m overburden and no subsidence factors considered, the passage of standard vehicles on the bridge does not affect the pipeline, but the passage of heavy vehicles results in stress levels that exceed the safety threshold.

With a 6 m overburden and considering a 10.4-ton vehicle load on the bridge (Comparison between scenario 3 and scenario 4), the addition of a 20 mm subsidence load increases the maximum stress in the pipeline system from 234.9 MPa to 302.1 MPa and the maximum stress at the girth weld increases from 166.7 MPa to 302.1 MPa. The pipeline’s maximum stress increases by 28.6% while the maximum stress at the girth weld increases by 81.2%. Multiple locations in the pipeline system show signs of failure, and the girth weld transitions from a safe state to a failure state.

Therefore, subsidence significantly increases the stress levels in the pipeline, causing stress concentration at the 12 o’clock position of the girth weld that exceeds the yield strength. This is a primary cause of the failure, consistent with the subsidence data observed during heavy rainfall in January as monitored by InSAR.

With a 6 m overburden and no vehicle load, the addition of a 20 mm subsidence load increases the maximum stress in the pipeline system from 206.6 MPa to 277.5 MPa (comparison between scenario 1 and scenario 7), and the maximum stress at the girth weld increases from 149.9 MPa to 277.5 MPa. The pipeline’s maximum stress increases by 34.3%, while the maximum stress at the girth weld increases by 85.1%. The girth weld transitions from a safe state to a failure state.

Therefore, subsidence significantly increases the stress levels in the pipeline, causing stress concentration at the 12 o’clock position of the girth weld that exceeds the yield strength.

## 4. Comprehensive Analysis

The results of the nondestructive testing on the girth weld indicate that there are numerous defects exceeding acceptable limits, rendering the weld quality non-compliant. Energy spectrum analysis of the inclusions within the weld joint reveals that their primary chemical elements are O, Si, Ti, Ca, and Al, which align with the main components of slag, indicating poor onsite welding quality of the girth weld. Additionally, microscopic observations show cracks propagating along the grayish material, where slag residue has disrupted the continuity of the weld joint, creating weak points in the girth weld.

The failed girth weld was designed to be buried 1 m deep. However, during its service life, the overlying soil gradually accumulated and increased, reaching a depth of 6 m at the time of failure, far exceeding the design depth. Stress analysis of the girth weld reveals that the increased burial depth does lead to an increase in stress at the top of the weld, but the increase in stress due to the added soil depth is relatively small, approximately 10%. However, a bridge located 10 m from the failed weld did not have protective casing during the pipeline installation. Analysis shows that the passage of heavy vehicles on the bridge causes a stress increase at the girth weld exceeding 20%, pushing it beyond the safety threshold. Furthermore, ground subsidence has significantly elevated the stress level in the pipeline, with the maximum stress increase at the girth weld exceeding 80%, greatly exacerbating stress concentration at the top of the weld and exceeding the yield strength.

Therefore, due to the poor welding quality of the girth weld, coupled with the increased soil cover, ground subsidence, and the impact of vehicle traffic on the nearby bridge, stress concentration at the girth weld, especially in areas with defects, was further aggravated, ultimately leading to the cracking and failure of the weld.

## 5. Conclusions and Recommendations

A girth weld in a pipeline experienced cracking, and failure analysis was conducted using experimental and finite element methods. The following conclusions can be drawn:

(1) The hardness test results of the submitted girth weld meet the requirements of GB/T 31032-2023 [28]. However, non-destructive testing revealed cracks, porosity, and lack of fusion defects, which do not meet the requirements of GB/T 31032-2023 [28]. No abnormal microstructures were found in the girth weld, but there were unmelted slag inclusions in the weld.
(2) The girth weld itself has poor welding quality. During use, the deepening of the overburden layer and ground subsidence exacerbated stress concentration at the girth weld, particularly at defect sites. As the load increased, cracks formed at the weld toe of the inner girth weld where welding defects were present. These cracks rapidly propagated along the defects, ultimately leading to the failure of the girth weld.

To prevent such failures from occurring again, the following measures are recommended:

(1) Strengthen on-site welding quality control of girth welds, conduct inspections for defects in girth welds of in-service pipelines, and promptly address any defects that exceed acceptable limits.
(2) In subsequent production and operation management, focus on monitoring the stress state at elbows No. 2 and No. 3, as well as the stress state of the pipeline crossing under the bridge. Install protective casing for the crossing if necessary.
(3) Utilize InSAR monitoring data to continuously monitor pipeline subsidence and use these data to conduct stress checks on the pipeline, particularly at the girth welds with phased array ultrasonic testing techniques, to prevent pipeline failure.

## Figures and Tables

**Table 1:** Vickers hardness test results.

Zone	Position	Hardness
Cap weld	5	151
Cap weld	6	154
Cap Weld	7	157
HAZ	8	178
HAZ	9	161
HAZ	10	157
Parent material	11	150
Transition weld	16	143
Transition weld	17	138
Transition weld	18	140
HAZ	19	167
HAZ	20	161
HAZ	21	153
Parent material	22	144
Root weld	27	153
Root weld	28	146
Root weld	29	147
HAZ	30	163
HAZ	31	169
HAZ	32	158
Parent material	33	145

**Table 2:** Considered scenarios.

Scenarios	Internal Pressure	Gravity	Vehicle Load	Subsidence	Pipeline Buried Depth
1	4.8 MPa	g = 9.8 m/s^2^	Ignoring	0 m	6 m
2	4.8 MPa	g = 9.8 m/s^2^	Bridge vehicle 49 t	0 m	6 m
3	4.8 MPa	g = 9.8 m/s^2^	Bridge vehicle 10.4 t	0 m	6 m
4	4.8 MPa	g = 9.8 m/s^2^	Bridge vehicle 10.4 t	0.02 m	6 m
5	4.8 MPa	g = 9.8 m/s^2^	Ignoring	0 m	1 m
6	4.8 MPa	g = 9.8 m/s^2^	Bridge vehicle 10.4 t	0 m	1 m
7	4.8 MPa	g = 9.8 m/s^2^	Ignoring	0.02 m	6 m

**Table 3:** Pipeline Mises stress calculation result.

Scenarios	Soil Depth (m)	Settlement (mm)	Vehicle Loads (t)	Internal Pressure (MPa)	Maximum Stress (MPa)	Maximum Stress Position	Failed Girth Weld Maximum Stress (MPa)	Girth Weld Failed or Not *
Scenario 1	6	0	0	4.8	206.6	Bend near the bridge	149.9	No
Scenario 2	6	0	49	4.8	304.3	Bend near the bridge and the pipe under the bridge	260.8	failed
Scenario 3	6	0	10.4	4.8	234.9	Bend near the bridge	166.7	No
Scenario 4	6	20	10.4	4.8	302.1	Girth weld	302.1	failed
Scenario 5	1	0	0	4.8	202.4	Bend near the bridge and the pipe under the bridge	136	No
Scenario 6	1	0	10.4	4.8	226.5	Bend under the bridge	150.5	No
Scenario 7	6	20	0	4.8	277.5	Girth weld	277.5	failed

*: The failure criteria was based on stress comparision.

## Data Availability

The raw data supporting the conclusions of this article will be made available by the authors on request.
